# Spontaneous Intracranial Hypotension Discovered Following Cerebral Venous Thrombosis: A Case Report and Review of the Literature

**DOI:** 10.7759/cureus.62884

**Published:** 2024-06-22

**Authors:** Yuma Hiratsuka, Yasufumi Ohtake, Mamoru Fukuda, Hirohiko Nakamura

**Affiliations:** 1 Department of Neurosurgery, Nakamura Memorial Hospital, Sapporo, JPN

**Keywords:** s-cerebral venous thrombosis, gadolinium-enhanced imaging, low-dose oral contraceptives, epidural blood patch, orthostatic headache, isolated cortical vein thrombosis, spontaneous intracranial hypotension

## Abstract

Cerebral venous thrombosis (CVT) is a rare complication of spontaneous intracranial hypotension (SIH). We encountered a case where SIH was discovered after the diagnosis of CVT, suggesting the occurrence of CVT during the acute phase of SIH. We report this rare case of isolated cortical vein thrombosis in the acute phase of SIH. A 48-year-old woman taking low-dose oral contraceptives presented with neck pain, headache, and right-sided weakness. Magnetic resonance imaging and digital subtraction angiography confirmed isolated cortical vein thrombosis. No other specific imaging abnormalities were noted. The patient was initially treated with anticoagulation. Subsequent worsening of her orthostatic headache led to the diagnosis of SIH, with diffuse dural enhancement on gadolinium-enhanced T1-weighted imaging. An epidural blood patch was performed, resulting in a favorable outcome with no neurological deficits. Although CVT can occur in the acute phase of SIH, particularly in patients with thrombophilia, the lack of characteristic imaging findings associated with SIH often complicates the diagnosis.

## Introduction

Spontaneous intracranial hypotension (SIH) is characterized by decreased intracranial pressure due to cerebrospinal fluid leakage. Affected patients develop symptoms such as orthostatic headache, neck pain, visual disturbances, dizziness, nausea, vomiting, and fatigue. Although subdural hematoma and cerebral superficial siderosis are known complications in patients with SIH, cerebral venous thrombosis (CVT) is rarely reported as a complication [1]. Furthermore, although CVT often develops several weeks after the onset of SIH, few reports have described such occurrences in the acute phase. We report a case in which SIH was discovered following the diagnosis of CVT. The patient was considered to have developed cortical venous thrombosis during the acute phase of SIH.

## Case presentation

**Figure 1:**
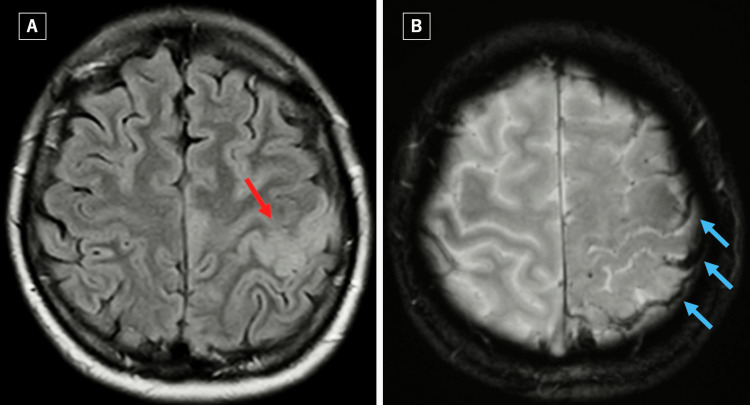
Fluid-attenuated inversion recovery (FLAIR) image shows cortical edema around the left central sulcus (red arrow) (A). T2*-weighted image shows cortical venous thrombi (blue arrows) (B).

**Figure 2:**
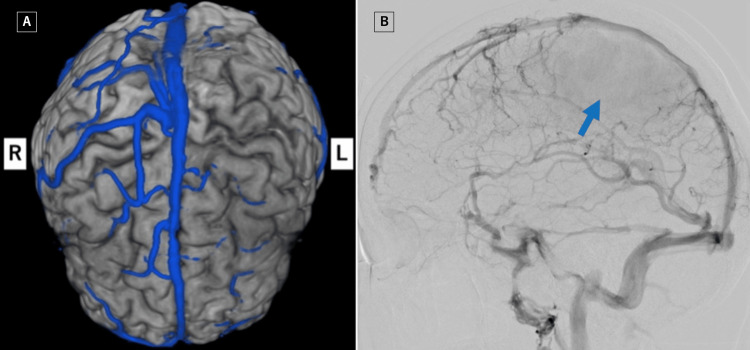
Magnetic resonance venography (A) and digital subtraction angiography (lateral view) (B) show occlusion of the left cerebral cortical veins, including the vein of Trolard (blue arrow).

A 48-year-old woman who had been diagnosed with dysmenorrhea and was taking low-dose oral contraceptives developed persistent neck pain and headache after nearly falling. Three days later, she experienced right-sided weakness and was admitted to our hospital. She underwent magnetic resonance imaging. Fluid-attenuated inversion recovery imaging revealed cortical edema in the left parietal lobe, while T2*-weighted imaging showed a thrombus in the cortical vein of the left hemisphere (Figure 1A, B). Magnetic resonance venography and digital subtraction angiography demonstrated occlusion of the superficial cerebral veins, including the vein of Trolard (Figures 2A, B). Blood tests revealed protein S activity of <10%. A diagnosis of isolated cortical vein thrombosis was made, and treatment with continuous heparin infusion and oral warfarin was initiated. Low-dose oral contraceptives were discontinued. The right-sided weakness subsequently improved, and the headache temporarily subsided.

**Figure 3:**
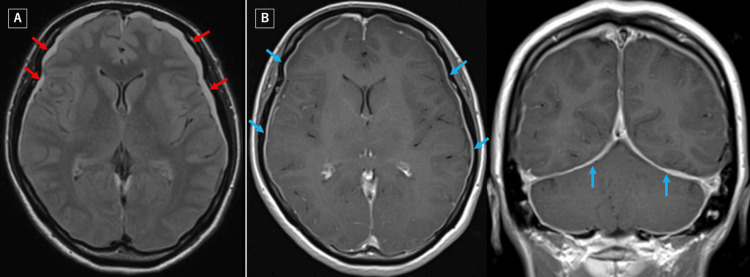
Fluid-attenuated inversion recovery (FLAIR) image shows bilateral subdural effusion (red arrow) (A). Gadolinium-enhanced T1-weighted images (axial and coronal views) show pachymeningeal enhancement (blue arrow) (B).

Follow-up magnetic resonance imaging showed improvement in cortical edema, and magnetic resonance venography demonstrated improved venous drainage. However, the patient experienced exacerbation of the headache on standing. Fluid-attenuated inversion recovery imaging revealed bilateral subdural effusion, and gadolinium-enhanced T1-weighted imaging showed diffuse dural enhancement (Figure 3A, B). Based on the presence of the orthostatic headache and the imaging findings, a diagnosis of SIH was made. Despite conservative management with bed rest and hydration, no improvement occurred. Therefore, we initiated treatment with an epidural blood patch and discontinued the anticoagulants. The orthostatic headache resolved postoperatively. Discontinuation of low-dose oral contraceptives led to an increase in the protein S activity to 53.6%. The patient fully recovered and was discharged home without anticoagulants. Subsequent follow-ups showed no recurrence of the headache. One month after discharge, magnetic resonance imaging revealed the resolution of the effusion and normal venous drainage.

## Discussion

Although subdural hematoma and cerebral superficial siderosis are known complications of SIH, CVT has rarely been reported as a complication [1]. In previous reports, SIH was diagnosed either after or concurrently with CVT. In the present case, however, SIH was discovered after the diagnosis of CVT, suggesting the occurrence of CVT during the acute phase of SIH. The mechanism by which CVT occurs in SIH is not fully understood, but several possibilities have been proposed. One possibility is that decreased intracranial pressure leads to venous dilation and decreased blood flow velocity, resulting in thrombosis [2,3]. Another possibility is that cerebral venous traction due to brain descent leads to venous injury, stenosis, or occlusion, resulting in thrombosis [3]. In this case, CVT occurred in the very early stages, before the detection of brain descent or subdural hematoma, suggesting that venous traction played a minor role. We presumed that the thrombosis resulted from decreased blood flow velocity due to venous dilation, which was possibly exacerbated by the patient’s use of low-dose oral contraceptives. The appropriate duration of anticoagulant therapy in such cases is a topic of debate. However, in patients without underlying thrombotic tendencies or when the causative factor can be eliminated, discontinuation of anticoagulants after resolution of venous occlusion and completion of SIH treatment may be feasible. In some cases, epidural blood patch alone has resulted in favorable outcomes [4], emphasizing the importance of treating the underlying SIH.

CVT as a complication of SIH is rare, with an incidence of only 2.1% [2]. Isolated cortical vein thrombosis is an even rarer complication at 0.4% [5]. We identified 53 previously reported cases, including the present case [2-18]. Among these 53 cases, thrombosis involving the cerebral venous sinuses was observed in 38 (71.7%) and isolated cortical vein thrombosis was observed in 15 (28.3%). Most patients develop CVT several weeks after the onset of SIH. Cases of CVT occurring in the acute phase of SIH (within 5 days of onset) have also been reported. In 6 (11.3%) of the 53 cases reported to date, including the present case, the patients developed CVT within 5 days of onset (Table 1). In two of these six cases (including the present case), the CVT occurred as early as 3 days after onset. Also among these six patients, two had isolated cortical vein thrombosis, only one had a subdural hematoma, and thrombotic tendencies were suggested in three. These findings suggest that CVT can occur very early in the course of SIH, particularly in patients with thrombotic tendencies.

**Table 1:** Clinical and radiological data on six patients with cerebral venous thrombosis within five days after onset of spontaneous intracranial hypotension NA: not available, F: female, M: male, MRI: magnetic resonance imaging, SIH: spontaneous intracranial hypotension, CVT: cerebral venous thrombosis, SDE: subdural effusion, SSS: superior sagittal sinus, TS: transverse sinus, SS: sigmoid sinus, JV: jugular vein, AC: anticoagulation, EBP: epidural blood patch, ICH: intracranial hemorrhage.

Reference	Age (years), sex	Interval from the onset of SIH to the diagnosis of CVT	Initial MRI (brain sag/SDE)	CVT location	Thrombophilia	Treatment	Complications
Rozen [6]	NA	5 days	NA/No	SSS, TS, SS, and proximal JV		AC	
Lin et al. [7]	43, F	5 days	Yes/No	Superficial cerebral veins	Protein S deficiency	AC/EBP	ICH
Huang [16]	29, F	3 days	NA/No	SSS		AC	
Pandey and Rooeintan [17]	42, F	5 days	Yes/Yes	SSS, TS, SS	mRNA COVID-19 vaccination	AC/EBP	
Li et al. [18]	37, M	5 days	Yes/No	SSS		AC/EBP	ICH
Present case	48, F	3 days	No/No	Superficial cerebral veins	Oral contraceptive	AC/EBP	

The CVT-associated mortality rate ranges from approximately 4% to 13%, with many cases resulting in incomplete recovery [19,20]. However, our patient had a favorable outcome. According to previous reports of patients with SIH, 81% of those with CVT involving the cerebral venous sinuses and 83.3% of those with CVT involving the cortical veins achieved full recovery; only one death was reported, indicating a relatively good prognosis [5,9]. This may be attributed to the resolution of intracranial hypotension through conservative treatments or an epidural blood patch, which alleviates venous dilation and traction, making thrombosis less likely to progress. However, a case of progression from cerebral cortical vein thrombosis to cerebral venous sinus thrombosis has been reported [2]. Therefore, caution is required.

In this case, there were no characteristic imaging findings of SIH at the initial consultation, making the diagnosis challenging. Although the co-occurrence of CVT during the acute phase of SIH is rare, diagnosis is often difficult because the typical imaging findings of SIH may not appear in such cases. Therefore, careful consideration of each individual case is important.

## Conclusions

We have reported a case of isolated cortical vein thrombosis in the acute phase of SIH, possibly triggered by thrombophilia associated with low-dose oral contraceptives. Although CVT can occur during the acute phase of SIH, especially in patients with underlying thrombotic factors, the lack of characteristic imaging findings associated with SIH often complicates the diagnosis. Therefore, detailed patient interviews and careful consideration are necessary. Additionally, treatment of SIH is as crucial as anticoagulation in managing CVT as a complication of SIH.
